# ANACONDA: a new tool to improve mortality and cause of death data

**DOI:** 10.1186/s12916-020-01521-0

**Published:** 2020-03-09

**Authors:** Lene Mikkelsen, Kim Moesgaard, Michael Hegnauer, Alan D. Lopez

**Affiliations:** 1 Melbourne School of Population and Global Health, The University of Melbourne, Carlton, Victoria 3053 Australia; 2 Institute of Public Health, Aarhus University, Aarhus, Denmark; 3 Swiss Tropical and Public Health Institute, University of Basel, Basel, Switzerland

**Keywords:** ANACONDA, Cause of death, Data, Mortality, Policy, Statistics, Quality

## Abstract

**Background:**

The need to monitor the Sustainable Development Goals (SDGs) and to have access to reliable and timely mortality data has created a strong demand in countries for tools that can assist them in this. ANACONDA (Analysis of National Causes of Death for Action) is a new tool developed for this purpose which allows countries to assess how accurate their mortality and cause of death are. Applying ANACONDA will increase confidence and capacity among data custodians in countries about their mortality data and will give them insight into quality problems that will assist the improvement process.

**Methods:**

ANACONDA builds on established epidemiological and demographic concepts to operationalise a series of 10 steps and numerous sub-steps to perform data checks. Extensive use is made of comparators to assess the plausibility of national mortality and cause of death statistics. The tool calculates a composite Vital Statistics Performance Index for Quality (VSPI(Q)) to measure how fit for purpose the data are. Extracts from analyses of country data are presented to show the types of outputs.

**Results:**

Each of the 10 steps provides insight into how well the current data is describing different aspects of the mortality situation in the country, e.g. who dies of what, the completeness of the reporting, and the amount and types of unusable cause of death codes. It further identifies the exact codes that should not be used by the certifying physicians and their frequency, which makes it possible to institute a focused correction procedure. Finally, the VSPI(Q) allows periodic monitoring of data quality improvements and identifies priorities for action to strengthen the Civil Registration and Vital Statistics (CRVS) system.

**Conclusions:**

ANACONDA has demonstrated the potential to dramatically improve knowledge about disease patterns as well as the functioning of CRVS systems and has served as a platform for galvanising wider CRVS reforms in countries.

## Background

More than a decade ago, *the Lancet* series ‘Who Counts?’ [1] drew attention to the fact that cause of death (COD) statistics from Civil Registration and Vital Statistics (CRVS) systems, despite being the main source of national and international COD information, often were of very poor quality, and, moreover, had improved very little over the previous half century. Only 31 countries representing 13% of the world population had at that time data that was considered to be of reasonably good quality and fit for purpose to inform public policy debates [2]. More recently, a global assessment [3] found that despite increasing demand for better quality data, particularly by the Millennium Development Goals (MDGs), and more recently, the Sustainable Development Goals (SDGs) [4], progress remains patchy.

The recent emphasis on monitoring progress has produced a few assessment tools [5] but none with a specific focus on the quality of the mortality collection system and the data produced. Mikkelsen et al. [3] in their global analysis of CRVS systems suggested that the Vital Statistics Performance Index (VSPI) [6] could yield useful insights into which components of the mortality system are most in need of improvement efforts. Their analysis also showed that those countries experiencing the most progress in a relatively short time were those in which there had been ‘sustained and informed government commitment’ [3], and where information and communications technologies were applied at the same time. This is important, since knowledge generated from vanguard countries can be used by others to advance their systems, particularly if technical leadership and advice about how to improve the defined system weaknesses is available to them.

The poor quality of global COD data is not only, as is often assumed, due to the high proportion of deaths occurring in the community, away from hospitals and physicians. A systematic review of studies investigating the quality of hospital death certificates [7] concluded that there were systematic and extensive misdiagnoses of COD by physicians in hospitals. The review covered studies undertaken in the period 1983–2013, and all showed a considerable degree of misclassification of COD, varying between 25 and 62% for those studies that used the International Statistical Classification of Diseases and Related Health Problems (ICD), Version 10 (ICD-10) at the three-digit level. Yet in many countries, hospital data are the only source of information about mortality patterns; hence, it is imperative that the data perform to the standards required to support good public policy.

There are a few well-documented strategies and practical interventions that countries can use to reduce uncertainty about what people die from. A series of recommended key actions that can help to overcome some of these challenges have been proposed in a recent policy brief for the Asia-Pacific Region [8]. Training of physicians in proper ICD COD certification as well as training of coders has been proposed in numerous studies [9, 10], and several online tools have been created over the years for both ICD certification and coding [11, 12], but little is known about their usage and impact. More recently, for non-hospital deaths, new automated verbal autopsy tools have been developed that allow large-scale application and provide reliable insight into COD pattern for community deaths [13, 14].

With most evaluations and proposed pathways for vital statistics development found in the literature focused on how complete and timely the registration/reporting of vital events are [5], a framework to facilitate quality improvement was proposed by the Health Information Knowledge Hub at the University of Queensland in 2010 [15]. This framework was later used by the World Health Organization (WHO) to develop an electronic assessment tool, analysing mortality levels and causes-of-death (ANACoD) [16]. While ANACoD proved to be a useful tool to analyse mortality and COD data, experience with the tool suggested that countries needed more guidance to usefully assess the key drivers of poor data quality in their systems.

To better meet policy need, a more sophisticated diagnostic data quality assessment tool has been developed and widely applied under the Bloomberg Data for Health (D4H) Initiative at the University of Melbourne (UoM) [17]. The tool is known as ANACONDA (Analysis of Causes of National Deaths for Action) [18]. This paper describes the methods, structure, and concepts underlying ANACONDA and reports on its application potential in countries to guide health information system developments to improve mortality and COD data quality.

The technical framework and software of ANACONDA are described in Additional file 1.

## Methods and standards used in ANACONDA

ANACONDA was developed to specifically assess the accuracy and completeness of mortality and COD data. It systematically takes users through a series of 10 steps and many sub-steps to perform arithmetic checks, calculate rates and indicators, and, importantly, facilitate comparison of country data with estimates based on the ongoing Global Burden of Disease (GBD) study. The tool was developed as a key platform for improving policy data for health through the strengthening of national mortality reporting systems. Through support from the D4H initiative, it has been possible to conduct both national and inter-regional ANACONDA trainings for countries to teach them how to use the tool and familiarise themselves with epidemiological and demographic concepts essential for mortality analysis.

ANACONDA is essentially built around the data quality dimensions which have been empirically identified by Philips et al. [6] to determine the performance of vital statistics systems globally in a standardised and detailed way. ANACONDA also includes a summary indicator for overall data quality as assessed by the five components that measure the overall quality of mortality data, labelled as the Vital Statistics Performance Index for Quality (VSPI(Q)).

ANACONDA uses global standards of disease classification, such as the International Statistical Classification for Diseases and Related Health Conditions and the GBD Classification, and applies common demographic and epidemiologic techniques and principles to assess the data. Each quality dimension is evaluated according to a series of specific analyses or sub-steps that interrogate the data, and calculate indicators or statistics that can be compared with similar data drawn from the UN population estimates, the Inter-agency Group for Child Mortality Estimation, and the GBD database. To apply ANACONDA, the input data must be entered with ICD-10 codes (3 or 4 digits) and compiled into 5-year age groups, except for deaths under age 5 which are disaggregated into those occurring less than 1 year of age and 1–4 years.

In the following sections, we will go through the ten steps of the ANACONDA analysis and show extracts of examples using real country data from the WHO Mortality Database for three unnamed countries from three regions of the world (Latin America, Africa, and Asia).

## Contents of the key ANACONDA steps

### Part I: All-cause mortality

#### Step 1: Age and sex structure of population and deaths

**Fig. 1:**
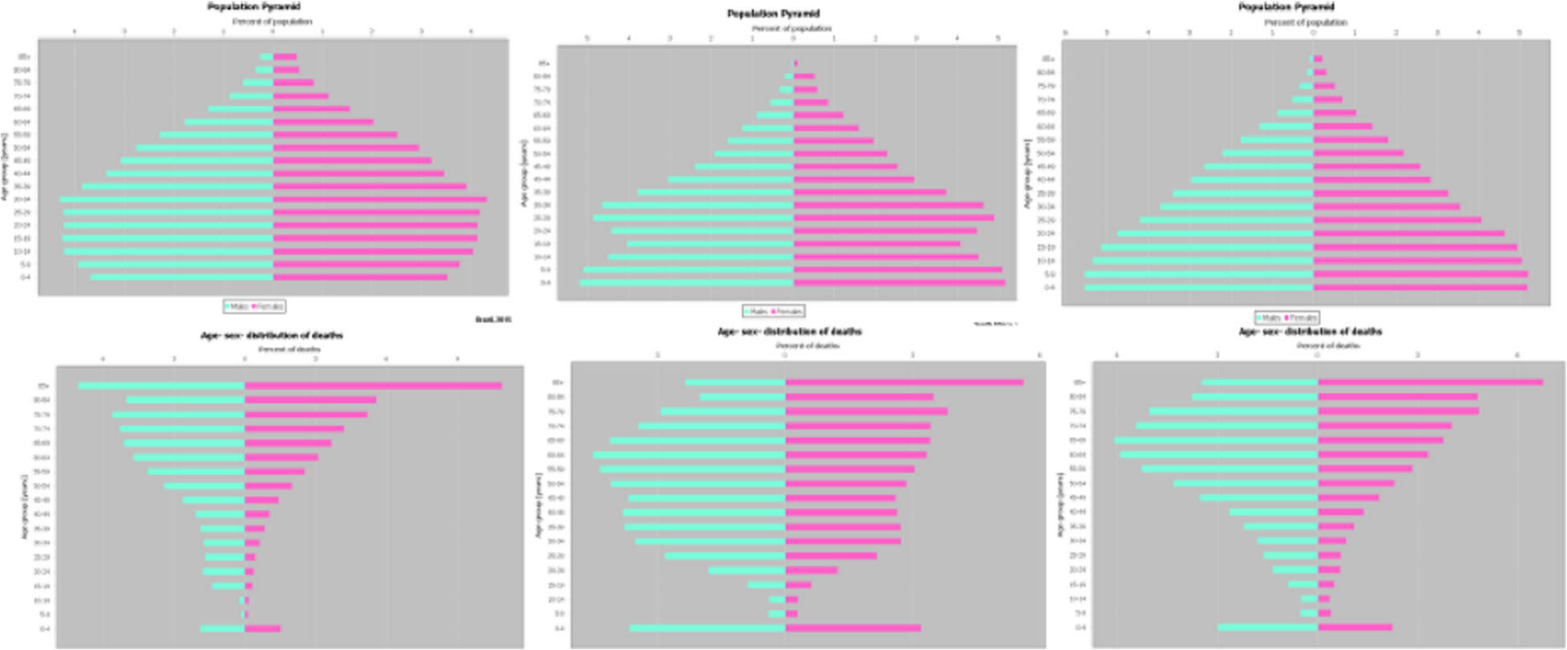
Examples of population age and death structure

The population age structure is crucial to correctly interpret mortality and COD data, since, irrespective of the health situation of a country, the age and sex structure of the population strongly influences the number of deaths. As a reflection of the strong age dependency of mortality rates, countries with older populations should expect, inter alia, higher crude death rates than countries with younger population age structures. ANACONDA produces both population and death pyramids that allow quick visual checks of the data as well as tables with absolute numbers and percentages. Comparator data facilitate further verification of the plausibility of the age structure of deaths and population, and the interpretation notes that accompany each step guide users in the interpretation of the data. Figure 1 shows three country examples of pyramid output clearly demonstrating the relationship between population ageing and the inverted pyramid structure showing the number of deaths at each age.

#### Step 2: Completeness of death reporting

The completeness of death reporting is closely related to how well the data represent the population they are supposed to describe. The crude death rate (CDR), a standard indicator of population health, can also be used to indicate the extent to which a vital registration system is able to capture all deaths.

**Fig. 2:**
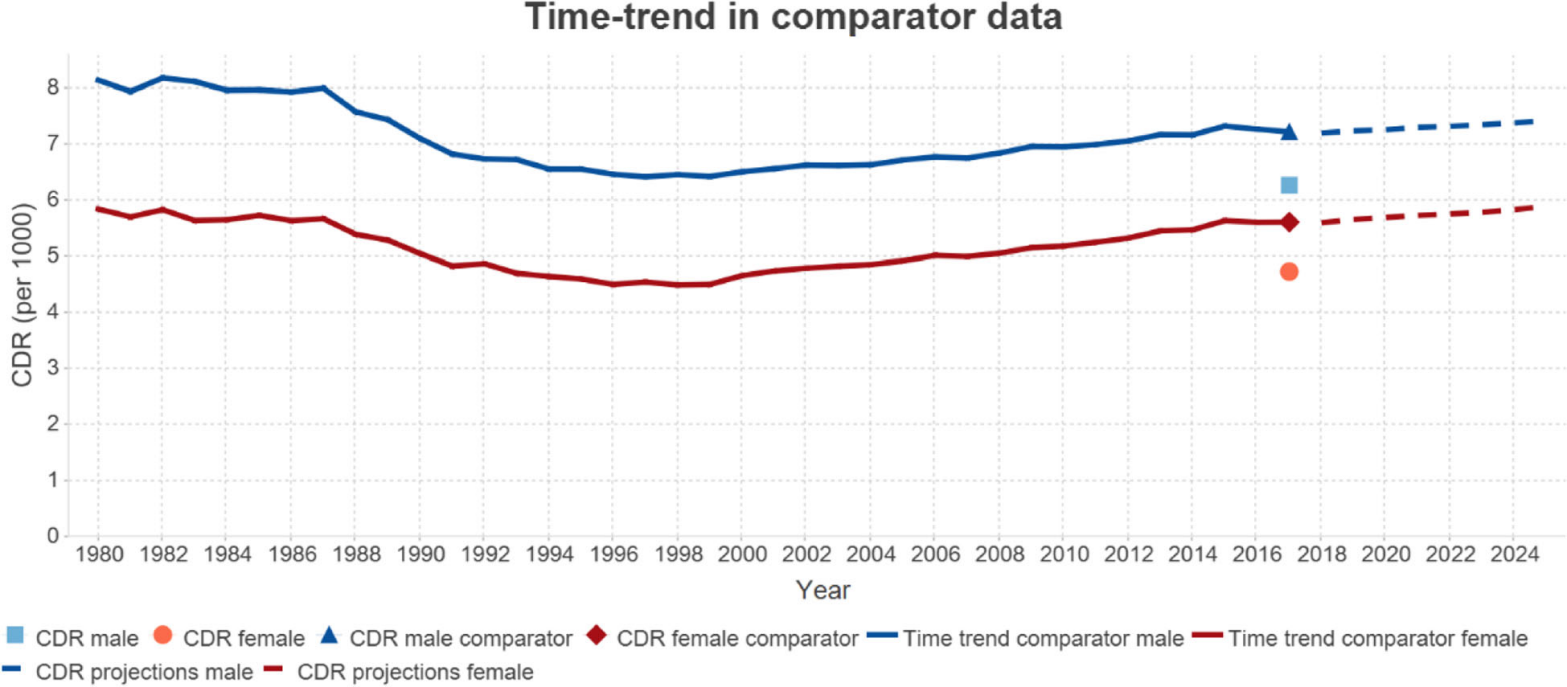
Crude death rate and time trend in the comparator data

ANACONDA uses two methods to assess completeness. First, from the entered data, the CDR is calculated and compared to the 30-year trend of estimated CDRs available in the GBD database for the given country. The GBD estimates the extent of under-reporting of deaths by applying standard death distribution methods such as the Generalized Growth Balance, Synthetic Extinct Generations, and a combination of the two, and then applies this adjustment factor to all adult deaths, by age, to estimate the number of adult deaths in each country-year [19]. Completeness of death registration for ages 5 and under is estimated by comparing under-five mortality estimates, derived from the vital registration system, to estimates of under-five mortality derived independently from censuses and surveys. A weighted average of these two age group estimates of completeness is computed to produce a single indicator of completeness, weighted according to the estimated number of deaths in each broad age group, which is then applied to the input data to estimate the ‘true’ crude death rate for any given country-year. ANACONDA shows this 30-year trend in the CDR from the GBD and compares the calculated (unadjusted) CDR from the input data to this trend (Fig. 2). The relative difference between the two measures of the CDR provides an estimate of the extent of under-registration of deaths.

The second method used in ANACONDA to assess completeness of death registration predicts completeness entirely from the input data, which greatly facilitates application of the method for estimating completeness of death registration at sub-national levels. The method is based on an empirical model of the relationship between the observed CDR and that which would have been predicted given the level of population ageing, the level of child mortality, and the completeness of child death registration in the country [20].

#### Steps 3 and 4: Consistency of age and sex reporting

**Fig. 3:**
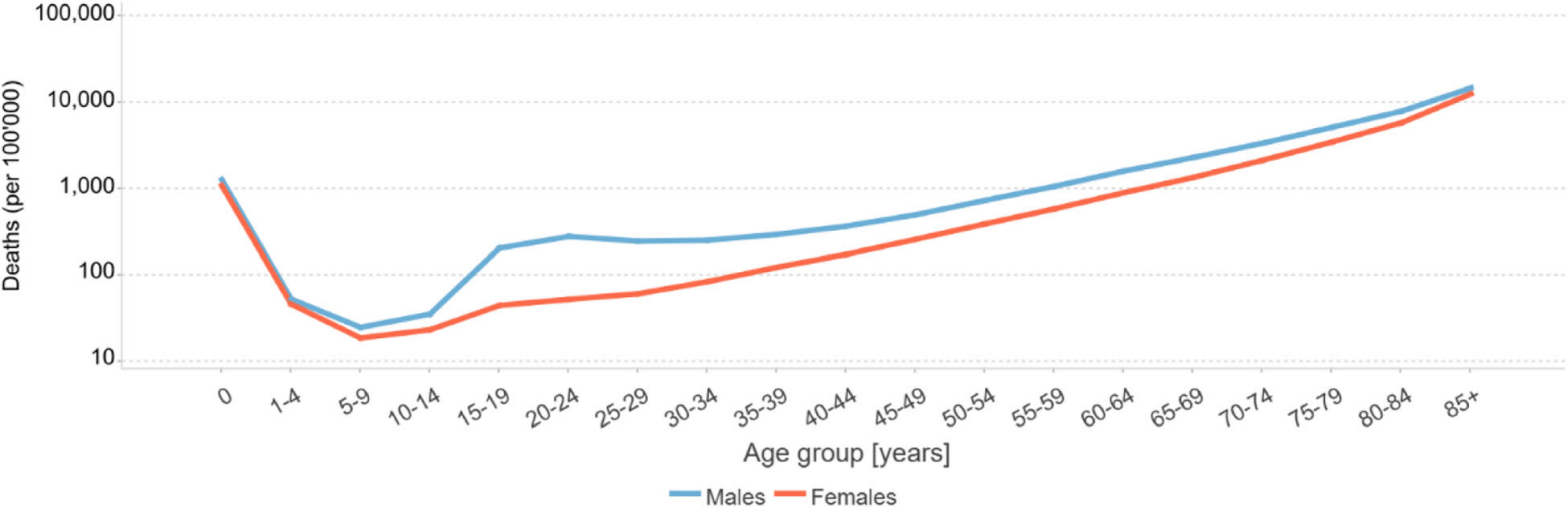
Age-specific mortality rates for males and females 2015

Mortality is closely related to ageing with death rates typically growing exponentially from around age 30. Deviations from this pattern are more easily checked by reviewing the natural log of the death rate which should increase linearly beyond about age 30, according to the Gompertz-Makeham law of mortality [21]. ANACONDA provides this visual check as shown in Fig. 3 for an example country. Further, the pattern of sex differences in mortality from the reported data can be readily assessed against the comparator data. This can be used to review, as an example, instances of excess female mortality which is extremely uncommon at any age.

#### Step 5: Child mortality

**Fig. 4:**
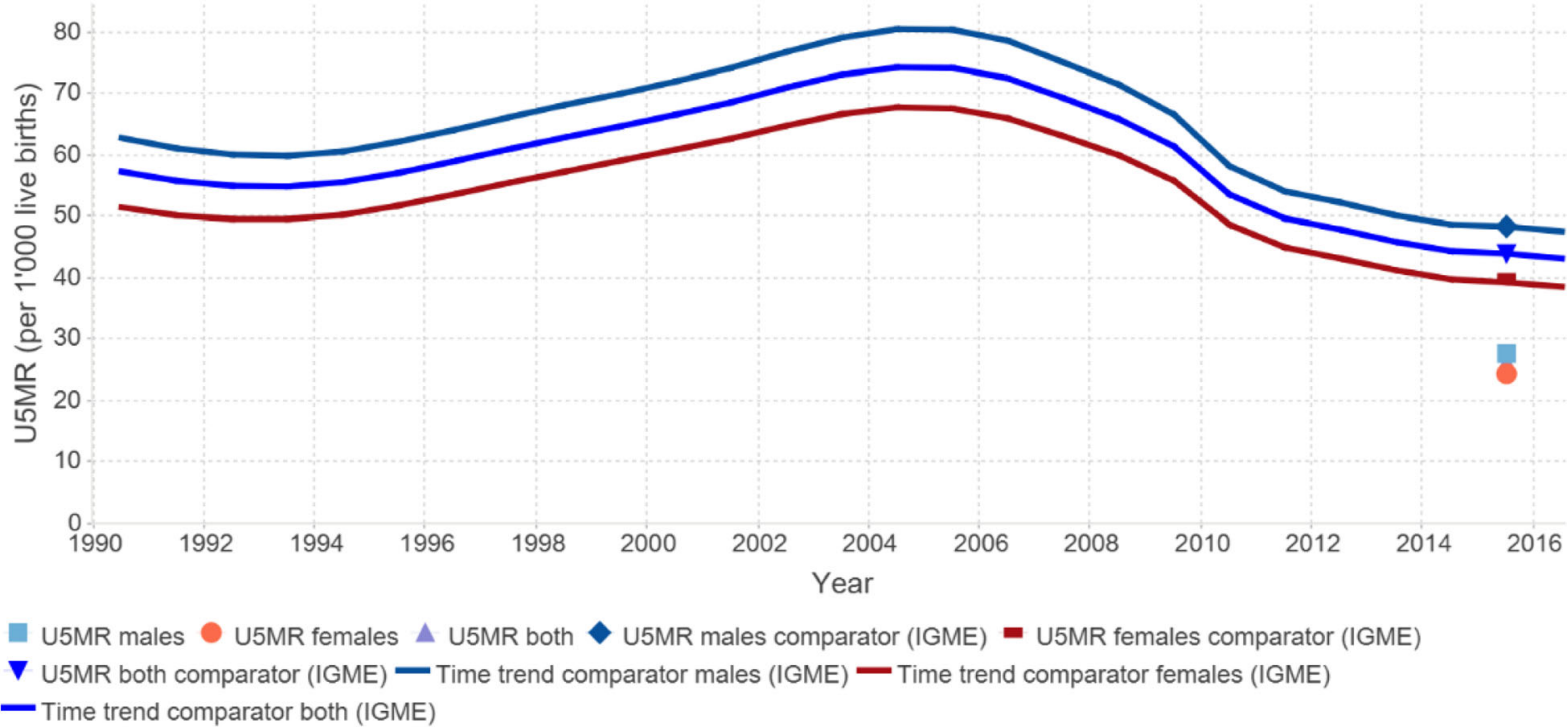
Child mortality indicator (5q0) 2015

Given the comparatively high mortality risks in the first few years of life, ANACONDA includes a specific focus on assessing how well deaths at these ages are being reported by calculating, from the input data, the probability of a child dying before its fifth birthday (5q0), which can be used to estimate the extent of under-registration of child deaths. Several studies have shown that child deaths, and particularly deaths in the first week, are more likely to be undercounted than deaths at any other age group [22, 23]. For countries that collect information on neonatal deaths, ANACONDA shows the proportion of all child deaths reported as early, late, or post neonatal deaths to better delineate the ages at which most child deaths are missed. These can then be examined and compared to the estimated deaths at each age from the GBD database. The extent of under-registration of child deaths is assessed by comparing the reported 5q0 with an estimated value that can be reliably calculated from the large amount of data generated from census and surveys undertaken in all countries. The UN Interagency Group for Child Mortality Estimation (IGME) provides such annual estimates for all countries. Figure 4 shows the probability of a child surviving to age 5 calculated by ANACONDA, based on the registered child deaths for 2015 for an example country, compared to the point on the best fit line provided by IGME for the country for the last 25 years.

### Part II: Causes of death

#### Steps 6, 7, and 8: Quality of cause of death reporting

Even in countries with complete or relatively complete recording/registration of deaths, the quality of data on the COD may not necessarily be reliable. According to the ICD, the COD that should be collected for statistical purposes is the one that led to the person dying, i.e. the underlying cause of death (UCOD). This distinction is important as it is not necessarily the same as the final condition or immediate or intermediate cause that led to death. If the physicians who certify the COD do not fill in the death certificate properly and record the appropriate UCOD, the information may be useless and will not serve its intended purpose.

**Fig. 5:**
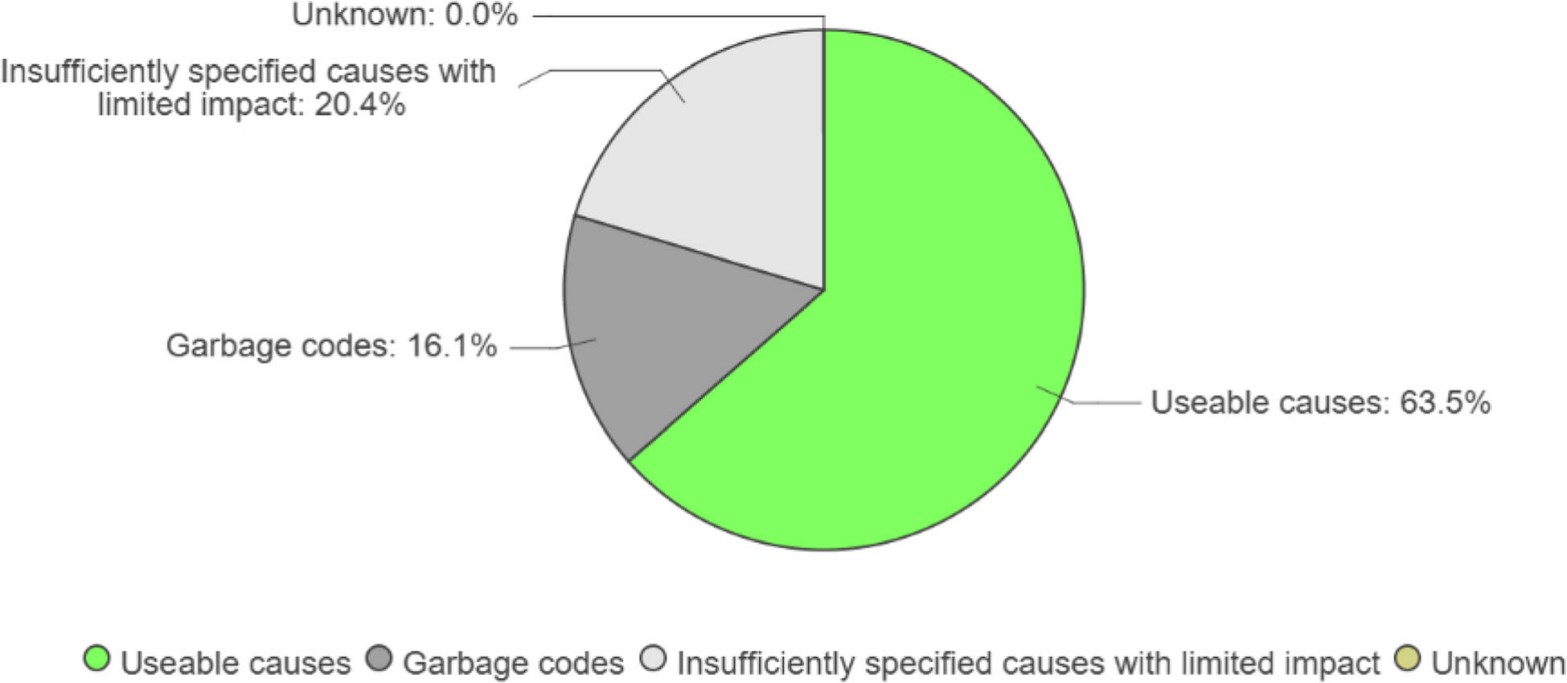
Distribution of causes of deaths by usability

ANACONDA provides a detailed framework for insight into the types of causes/codes in the input data that should be avoided because they cannot be a plausible UCOD and which therefore should be avoided as they have no or very limited public policy utility. As a first step, the usability of the input data is assessed by showing the fraction of codes that are *usable for public health purposes*, those that are considered *unusable* for public health purposes, and those that are *poorly specified*, as shown in Fig. 5.

**Fig. 6:**
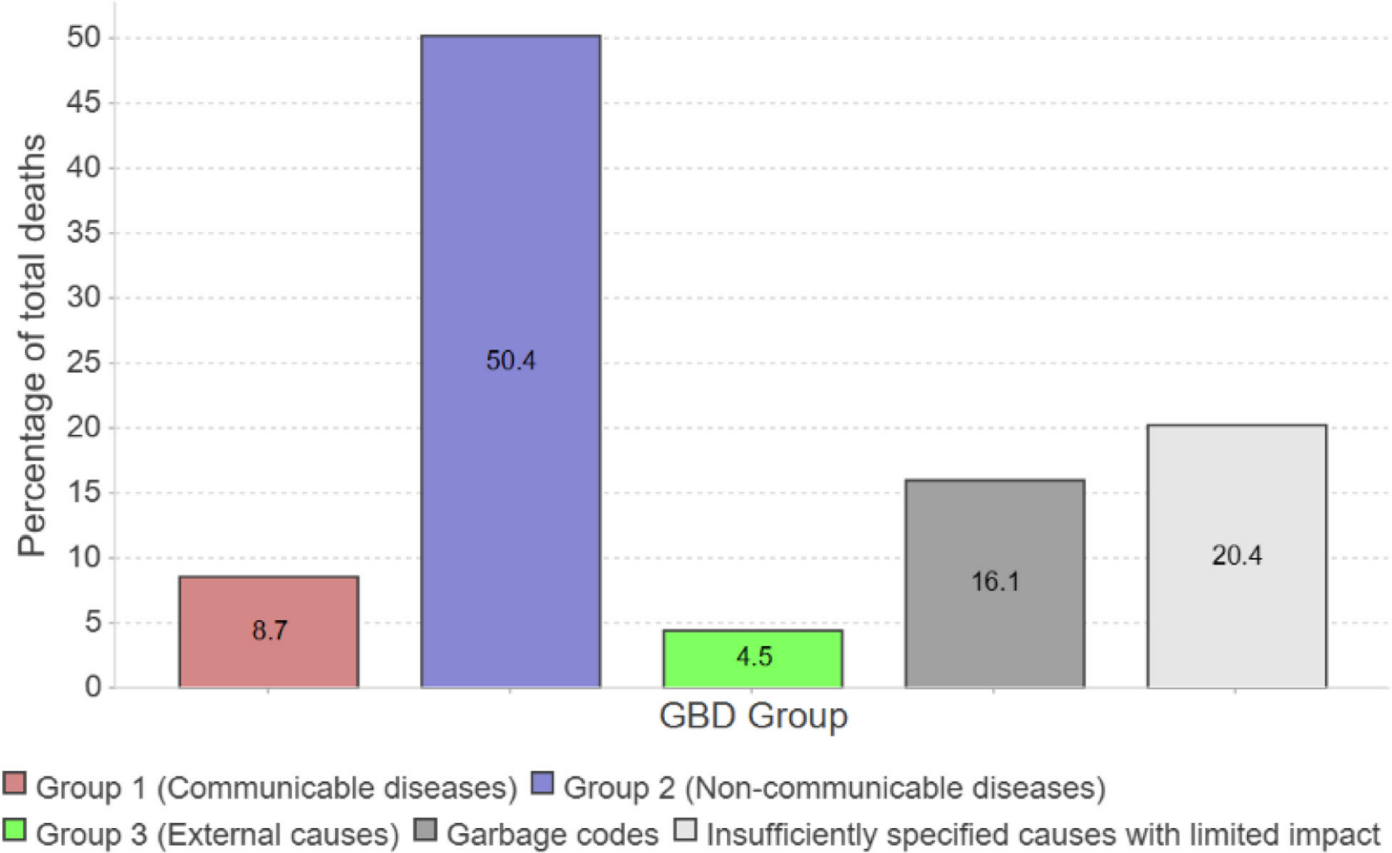
Cause of death distribution on broad cause groups, and unusable and poorly specified causes

Another key step in a COD quality assessment is to investigate the distribution of deaths on the three broad GBD disease groups ((1) communicable diseases: infectious and parasitic diseases, maternal and neonatal causes, and malnutrition; (2) non-communicable diseases including mental health; and (3) external causes: accidents, homicide, suicide, war, and natural disasters) to assess whether the observed pattern corresponds to what could be expected given information about the country’s epidemiological transition. Figure 6 shows how ANACONDA distributes the deaths across the three broad groups of health conditions and classifies the remainder, referred to as ‘garbage codes’,^1^ into ‘unusable causes’ and ‘insufficiently specified codes’. ANACONDA further calculates the relationship between the first two of the three broad GBD cause of death groups and compares this to what IHME predicts for the country as a measure of progress through the stages of epidemiological transition. The higher the proportions of the two types of garbage codes, particularly the unusable cases, the higher the likelihood that the dataset is of poor quality, with serious implications for misinforming policy debates about health priorities and resource allocation.

**Fig. 7:**
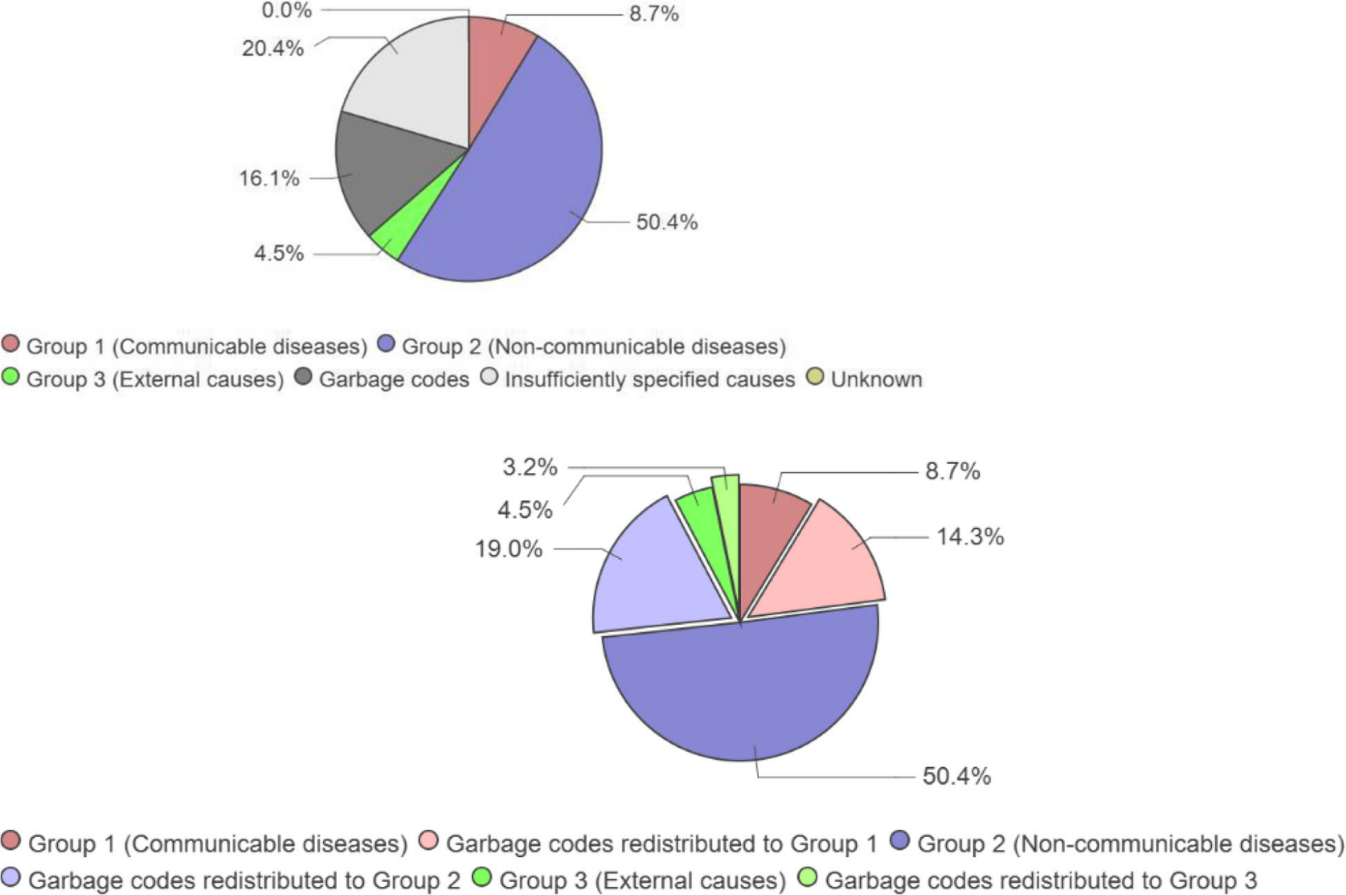
Input cause of death data and probable distribution of the unusable and poorly specified causes

ANACONDA further provides a comparison of the distribution of deaths across the three broad cause groups, based on the input data, to what might be expected after garbage codes have been redistributed according to established GBD algorithms [22], and added to the three groups. Figure 7 illustrates for a country the significant changes to sizes of the three broad disease groups as a result of allocating the garbage codes to valid cause groups, reminding policymakers of the need to proceed with caution when using data with high proportions of unusable and poorly specified codes.

To reduce the amount of garbage codes in the data, a better understanding of what these codes are and their frequencies is needed. ANACONDA provides suggestions to that insight by classifying all the uninformative and poorly specified causes into two different typologies—both offering valuable intelligence of the data. The first typology, based on the work by Lozano et al. [23], extracts all the garbage causes and classifies these into the following five groups based on ICD concepts: (1) symptoms, sign, and ill-defined conditions; (2) impossible as underlying causes of death; (3) intermediate causes of death; (4) immediate causes of death; and (5) insufficiently specified causes of death.

This classification, as it is based on the type of ICD error, provides insight into the extent to which physicians and others who certify deaths are knowledgeable about correct death certification practices by specifically showing the amount of each ICD error category in the data. While it may be argued that the fifth category in the classification contains causes that are not incorrect as a COD, they do however indicate that the certifier did not take the time to select a more precise diagnosis or perhaps did not understand the importance of accurately diagnosing the cause for planning and health policy purposes.

The second typology offered by ANACONDA identifies the causes of death that should not be used on the death certificate,^2^ and classifies these into four impact levels, thereby providing guidance as to where efforts to eliminate these ‘garbage codes’ should be concentrated (Table 1). For each level, the most commonly used unusable codes are extracted to allow elimination strategies to focus on these. Through a hierarchical process based on grouping similar garbage codes into packages at each of the four levels, the packages are ranked in order of importance, and the actual ICD codes which are most frequently used within each package are identified so that users can immediately see what practices are causing the highest amount of garbage codes. Table 2 shows the contents of the Sepsis package for one country. It is this detailed information that is likely to be most useful in guiding improvement strategies for quality of COD reporting.

**Table 1:** Typology of garbage codes based on severity of impact level for policy

1. **Level 1 (very high)—codes with serious policy implications**. These are causes for which the true UCOD could belong to more than one broad cause group, for example, septicaemia. Such errors can potentially grossly misinform understanding of the extent of an epidemiological transition in a population. 2. **Level 2 (high)—codes with substantial implications**. These are causes for which the true UCOD is likely to belong to only one or two of the three broad groups (i.e. ‘essential (primary) hypertension’). While not altering the understanding of the broad composition of mortality in a population, these codes can significantly affect the comparative importance of leading causes within broad disease categories. 3. **Level 3 (medium)—codes with important implications**. These are causes for which the true underlying UCOD is likely to be within the same ICD chapter. For instance, ‘unspecified cancer’ still identifies the death as being due to cancer and thus has some policy value, although greater type (site) specificity is required as different strategies are applied for different sites of cancer (i.e. breast versus lung). 4. **Level 4 (low)—codes with limited implications**. These are diagnoses for which the true UCOD is likely to be confined to a single disease or injury category (e.g. unspecified stroke would still be assigned as a stroke death). The implications of unusable causes classified at this level will therefore generally be much less important for public policy.

**Table 2:** Example of the contents of the Sepsis package of unusable codes

Rank	ICD code	Name of category	Total causes
1	A41	Other septicaemia	6547
2	D65	Disseminated intravascular coagulation	242
3	R02	Gangrene, not elsewhere classified	10
4	A40	Streptococcal septicaemia	5

**Fig. 8:**
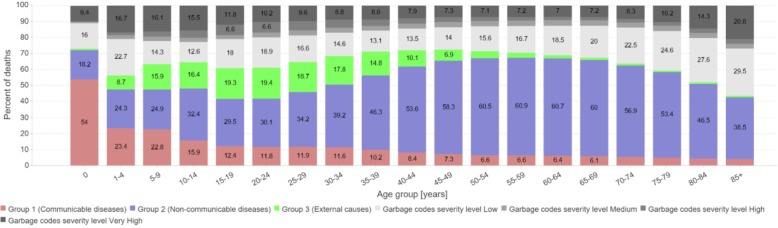
Age distribution on broad disease groups and distribution of garbage categories by age

For effective health promotion interventions, it is useful to know the age pattern of death within each of the broad COD groups. ANACONDA displays this in step 8 for the input data together with the amount of garbage codes found for each age group. From decades of epidemiological research, the disease age patterns are known and can be used as comparators (Fig. 8). In general, by far, the highest share of communicable diseases is found in children, while for non-communicable diseases, it is in the age groups 40 and above. For ages 15–30, the highest proportions of deaths are due to accidents and injuries; these patterns should be reflected in the data.

#### Step 9: Leading causes of disease

**Fig. 9:**
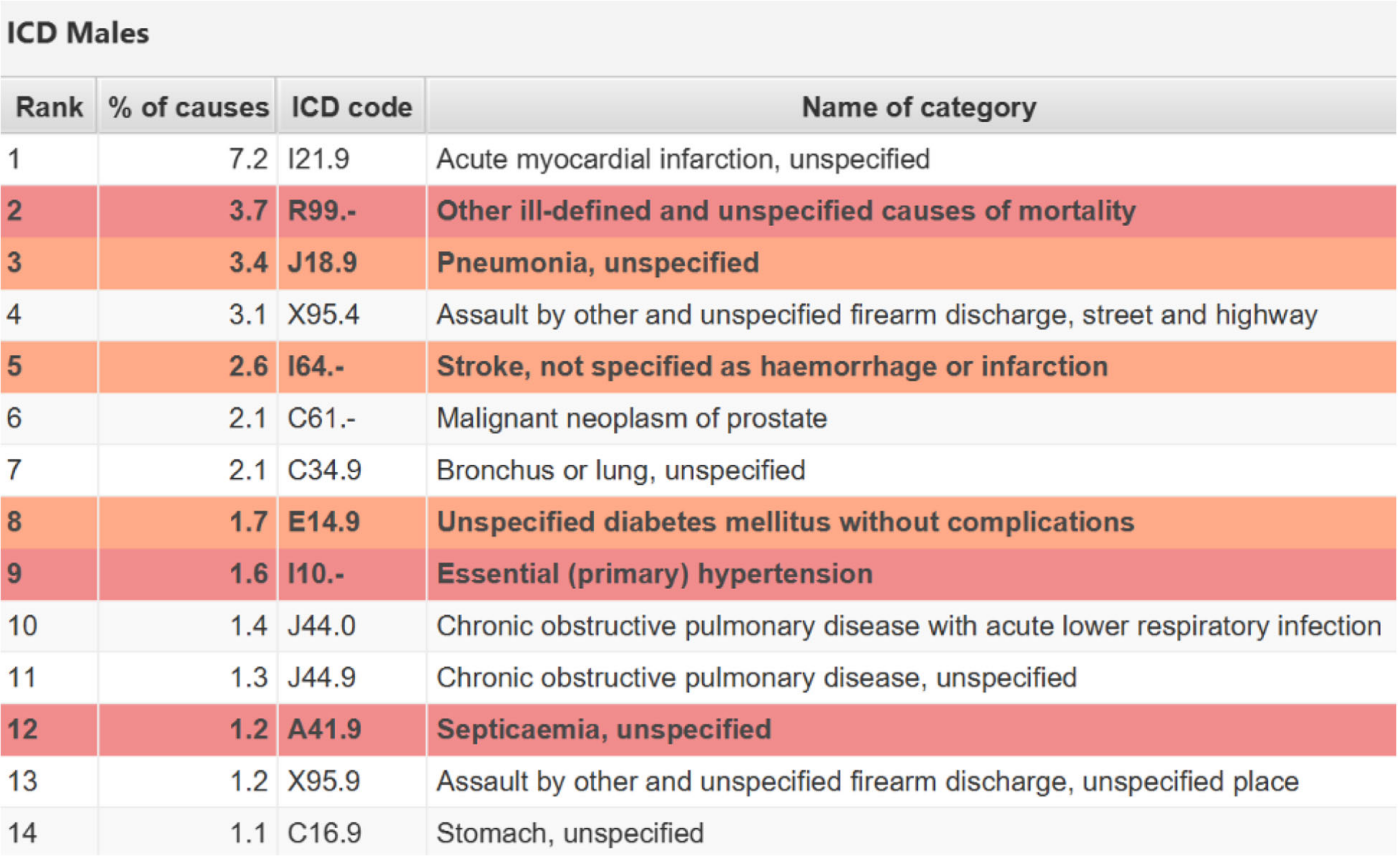
Leading causes of death

All health information systems should, as a bare minimum, be able to produce a table showing the leading causes of death for the population to guide health policy and priority-setting. If uninformative causes are found among the 20 leading causes identified by ANACONDA in the input dataset, this is an indication that the dataset is partially unreliable and not fit for many policy purposes. ANACONDA uses red to indicate garbage code categories that have the most impact on misguiding policy, and those of lesser policy consequence in orange (see Fig. 9 for a country example). ANACONDA also provides a comparator country dataset consisting of the input data compiled according to the GBD classification with the garbage codes shown separately, and another set with the garbage codes redistributed to the 20 leading causes according to complex country-specific algorithms. The aim should be to reduce and minimise the difference between the two datasets.

### Part III: System performance indicator

#### Step 10: the Vital Statistics Performance Index for Quality

The final step in ANACONDA is the calculation of a composite quality indicator of the input data, the VSPI(Q). This index is an adaptation of the VSPI developed by Philips et al. [6] but based solely on the quality components, where the timeliness dimension has been dropped. It provides one single summary score of the performance of the death reporting system based on its output. The score considers the essential components of data quality and weights these according to their importance in affecting the overall utility of the data for policy development. The five components that make up the index are as follows:

1. Completeness of death registration
2. Fraction of ‘garbage’ codes in the data
3. Amount of COD detail in the COD list used
4. Extent to which age and sex of deceased are not recorded
5. Number of biologically implausible causes found in the data (i.e. highly unlikely of impossible causes for a given age-sex category)

Regarding the weighting for the garbage codes at each level, we arbitrarily assumed, following Philips et al. [6], that severity levels 1–3 garbage codes were, on average, only half as informative as codes classified to level 4, and hence, these were penalised twice as much in the overall VSPI(Q) score.

For policy purposes, the greater the amount of granularity in the COD list, the more useful the data are likely to be. To score this component, the number of distinct causes of death reported in the dataset was compared to a standard reference list of 192 causes developed for the GBD study and considered as the minimum universe of causes which are of substantial public health interest [6]. The score was calculated by computing the proportion of the 192 GBD-standard causes which were available in the input data. The scores for the two remaining components were simply calculated as proportions.

**Fig. 10:**
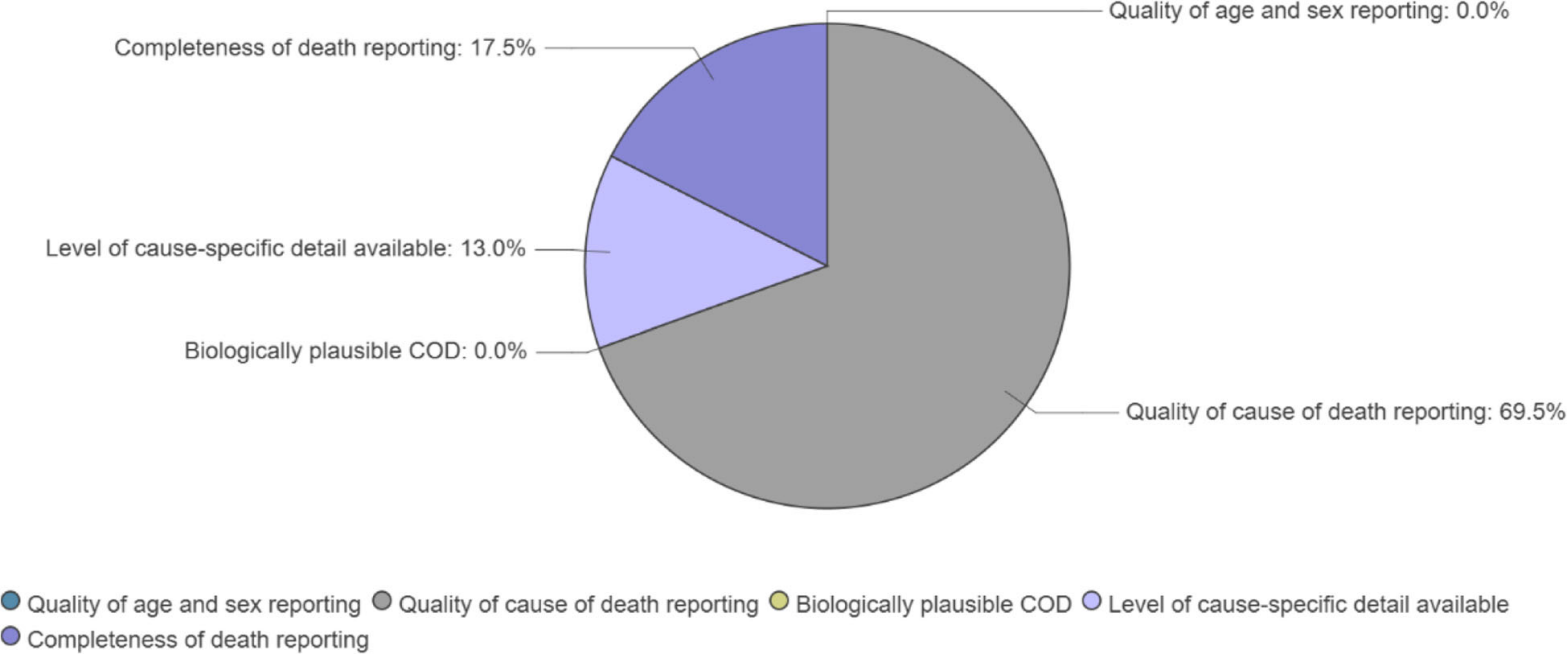
Priority areas for action for a country

ANACONDA weights and scores each of the five components based on the input data and transforms the scores according to their simulated impact on the actual or true cause-specific mortality fractions (CSMFs), taken as a measure of the overall policy utility of the data [6]. These transformed scores are then multiplied to arrive at the overall summary score of data quality, ranging from 0 to 100%. To assist countries to focus their improvement efforts, ANACONDA also provides a convenient visual graphic of the main contributors to the gap between the observed VSPI(Q) score and the maximum attainable (100%) (Fig. 10 shows an example country).

## ANACONDA usage in countries

Building a tool like ANACONDA for global use is a long process of development, testing, and feedback. The tool has therefore not been publicly released on the CRVS Knowledge Gateway [24] of the University of Melbourne, but has been widely disseminated through training workshops. Several of the 36 countries that have benefitted from the ANACONDA workshops have already, like the Philippines and Brazil, integrated the tool into their annual data production process and use it for checking and monitoring the quality of their data. Some countries are now themselves conducting trainings of regional staff in using ANACONDA (Brazil, China, Colombia, Peru, Philippines) thereby giving local authorities an understanding of problems in their data. Increasing local awareness of quality issues in the data is crucial, since it is at this level where most of the corrective action is needed to achieve overall improvement. In China, ANACONDA has specifically been used to train officials in mortality analysis, with these officials now able to show provincial authorities the flaws in their data and how to solve these. The introduction of ANACONDA through regional workshops with WHO, the United Nations Economic and Social Commission for Asia and the Pacific (ESCAP), and Economic Commission for Africa (ECA) has significantly expanded its use and has led to the initiation of related improvement efforts in medical certification and the recording of community deaths in several countries, among them Egypt, Iran, and Thailand. Given the demand for ANACONDA, we expect that the tool will be made publicly available at the CRVS Knowledge Gateway [24] before the end of the year.

## Discussion

For statistical offices and other data producers preparing annual reports of vital statistics or inputs for burden of disease studies [25], ANACONDA has much to offer in the form of checking the data for errors and inconsistencies, calculation of common mortality indicators, and numerous charts and figures. The figures and tables produced by ANACONDA can easily be exported as well as the cleaned data for further manipulation outside of the tool. A prefilled template available at the CRVS Knowledge Gateway [24] makes it possible to produce a comprehensive annual mortality report from the output with minimal efforts. Those who use ANACONDA regularly will appreciate the in-built monitoring function in the form of the VSPI(Q) that indicates whether their mortality system is improving or not changing at all, or whether they are managing to register more deaths and missing less child deaths.

**Fig. 11:**
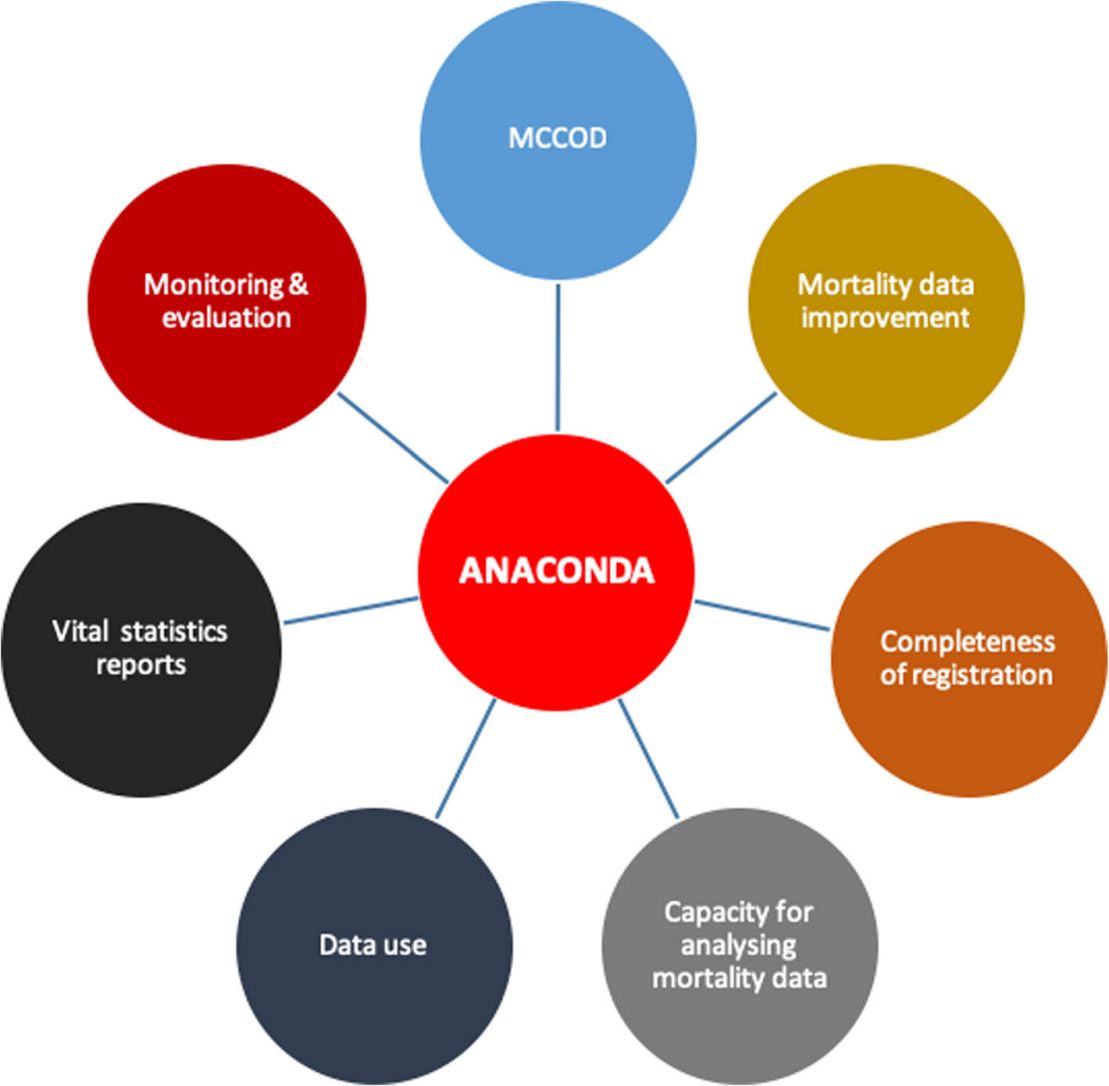
The ANACONDA platform for mortality system improvement

Although the main function of ANACONDA is to allow countries to comprehensively assess the accuracy and completeness of their mortality and COD data, it can be used for many other purposes (Fig. 11). Apart from the user guide and resources integrated into the tool, a guidance manual for assessing and interpreting mortality data with ANACONDA is available to download through the CRVS Knowledge Gateway [24]. Based on the evaluations from such trainings, conducted in many countries under D4H, participants who received this instruction built capacity to apply basic epidemiological and demographic concepts for analysing their datasets and conducting mortality analysis.

ANACONDA can also be used to inform the trainings of physicians in correct medical certification. For each country, it is possible to extract error patterns in certification and identify the exact codes that physicians misuse. This detailed assessment of medical certification is very valuable since it is known that country practices differ, and hence, a training that specifically incorporates country-specific issues is likely to be more successful in changing physician’s certification practices.

ANACONDA is very useful for those countries interested in measuring their mortality burden. The tool allows countries to carry out a detailed and comprehensive audit of their mortality data, which is critical if subsequent estimates of the burden of disease are to be correctly interpreted for policy purposes.

For countries and users considering the application of ANACONDA, there are a few limitations to keep in mind. The tool was developed with national datasets in mind, and therefore, small datasets, typically from hospitals for which no population at risk data exist, the steps involving the calculation of rates will not work. In addition, with less than 4000 deaths annually, some of the charts may have gaps due to small numbers for some age groups. Users should also be mindful that the analysis of garbage codes is based on the concept of the underlying COD; hence, if reported causes are taken from hospital discharge forms, the garbage code analysis will not be as informative as this is not the underlying cause (generally only reported on the death certificate). Because the COD data for ANACONDA must be entered with an ICD code, it is of limited use for VA data derived from the application of automated diagnostic methods since their cause lists do not provide individual codes. Perhaps more importantly, the comparators used in the tool mostly come from the GBD study, which attempts to estimate the likely true age-sex-cause pattern of mortality in a country after correcting for under-registration and garbage codes. The accuracy of the comparators is therefore likely to vary from country to country, and over time, depending on the amount of and reliability of the data and other information that was available on mortality and cause of death patterns for a country.

## Conclusions

ANACONDA has already proved to be a very popular tool for countries to assess the quality of their mortality data—in part because it does more than quality analysis. By making the tool user-friendly and explaining the rationale and objectives of the various steps, users can quickly appreciate the importance of identifying and monitoring data quality and errors. The tool empowers users to become activists for better data, to interact more effectively with medical associations and medical schools, and to apply innovative and generally cost-effective methods to increase death registration completeness. Countries spend very substantial sums of money each year on maintaining and expanding their CRVS systems; it is thus important that the data outputs from those systems represent value for money and are fit for purpose. ANACONDA provides the empirical evidence to ensure this accountability.

## Supplementary information

**Additional file 1.** Software architecture, building blocks, and resources included in ANACONDA.

## Data Availability

Not applicable.

## Abbreviations

ANACoD: Analysing mortality levels and causes of death
ANACONDA: Analysis of National Causes of Death for Action
CDR: Crude death rate
COD: Cause of death
CRVS: Civil Registration and Vital Statistics
ECA: United Nations Economic Commission for Africa
ESCAP: United Nations Economic and Social Commission for Asia and the Pacific
GBD: Global Burden of Disease
ICD: International Classification of Diseases and Related Health Problems
ICD-10: International Classification of Diseases and Related Health Problems–Version 10
ICT: Information and communications technology
IGME: Interagency Group for Child Mortality Estimation
IHME: Institute for Health Metrics and Evaluation
MDGs: Millenium Development Goals
SDGs: Sustainable Development Goals
UCOD: Underlying cause of death
VSPI: Vital Statistics Performance Index
VSPI(Q): Vital Statistics Performance Index for Quality
WHO: World Health Organization

## Footnotes

1 Term used in the first GBD study [18] to describe codes that have no or little utility for policy

2 See *Improving the quality of cause of death data for public health policy: are all ‘garbage’ codes equally problematic?* paper in this series.
