# Supplementary material for: ANACONDA: a new tool to improve mortality and cause of death data
Source: BMC Med. 2020 Mar 9;18:61. doi: 10.1186/s12916-020-01521-0 (PMC7061487; doi:10.1186/s12916-020-01521-0)
Supplement: Supplementary file 1 — Additional file 1. Software architecture, building blocks, and resources included in ANACONDA. [file 12916_2020_1521_MOESM1_ESM.docx]

**Software architecture, building blocks, and resources included in ANACONDA**

The technical framework and software of ANACONDA (Analysis of National Causes of Death for Action) has been developed with the aim to provide a user-friendly and easy to install piece of software based on standard technology supporting many platforms and heterogeneous environments. By packaging all required resources and software pre-requisites within the tool, there is no need for a complex information technology (IT) infrastructure and setup for users to run the software, nor is it necessary to connect to the internet.

ANACONDA is based on Java/JavaFX technology and its architecture has been designed to be flexible and extensible, allowing for continuous innovation and adaptation. The software is modularly structured consisting of various components such as; the data import module, the graphical user interface framework, the core domain model, the persistence layer and the data export functional module.

Data are entered in a standard Excel data input template available with the software. The import process into ANACONDA is facilitated by a graphical wizard supporting the user, while setting global options and reviewing the structure and range of the mortality and population data. Validity checks are performed on the input data to detect inconsistencies or missing data before saving the data in a proprietary ANACONDA format (mqr). This file can then be opened on any other system running ANACONDA to continue analysis or review the comments of another user.

The core of the software consists of a domain model encapsulating the specific functionality for the various analysis steps and sub-steps: aggregation of data, calculation of rates and indicators, performance of arithmetic and consistency checks, and comparison of input data or a summary measure of them with other sources of demographic and epidemiological estimates for a country or a geographic region. To do this, the software makes use of a wide variety of resources, codes and mapping files as well as comparator data, which are loaded dynamically where and when needed. A detailed listing of the resources and comparator data used by ANACONDA can be found in the appendix of this paper.

The graphical user interface is built on top of the domain model and allows the user to easily navigate through and interpret each analysis step and sub-step by providing graphical output and visualisations in tables, graphs and charts. For each step and sub-step, the user can review the results, input comments and assign an overall status with respect to the quality of the data. A data quality summary report will then be generated based on this review, which can be printed and used as a key element along with the summary Vital Statistics Performance Index for Quality (VSPI(Q)) to monitor progress.

The software also allows users to re-export the mortality and population data into various structured text formats or Excel. Summary tables calculated by the tool can be copied into Excel for further processing and charts can be exported and saved for presentations and use in other external programs.

An extensive set of introductory texts, background information and technical documentation are available as an integral part of the tool. The documents are directly accessible in the tool by navigating through a tree-like structure in a browser window which provides an overview of all available documentation. Under each step in the tool the information button provides users with further insight into the specific analysis step.

ANACONDA currently provides language packs in English, Portuguese, Spanish, Chinese and Russian. The language packs include translations for all control and visualisation elements contained in the graphical user interface, as well as for the entire documentation set.

To assist users to interpret their data and understand the calculations, a guidance manual is available to download in English (<https://crvsgateway.info/file/10084/56>), Chinese (<https://crvsgateway.info/file/9792/55>), Russian (<https://crvsgateway.info/file/10085/3022>), and Spanish (<https://crvsgateway.info/file/10756/65>). To further assist users, a prefilled report template is also available in English (<https://crvsgateway.info/file/10035/2812>) and Portuguese (<https://crvsgateway.info/file/10747/2606>), which enables users to produce an annual mortality report with minimum input by simply exporting the many outputs produced by ANACONDA.
